# Campus outdoor environment, learning engagement, and the mental health of college students during the COVID-19 pandemic: From the perspective of students in different grades

**DOI:** 10.3389/fpubh.2023.1143635

**Published:** 2023-04-11

**Authors:** Ning Sun, Wanting Liu, Zhenhua Zheng

**Affiliations:** College of Communication and Art Design, University of Shanghai for Science and Technology, Shanghai, China

**Keywords:** mental health, perceived campus outdoor environment, learning engagement, college students, group differences

## Abstract

**Introduction:**

During COVID-19, the mental health of Chinese university students has been a pressing concern. But the internal mechanism of perceived campus outdoor environment and learning engagement affecting college students’ mental health during the COVID-19 pandemic has not been fully discussed.

**Methods:**

The current study used cross-sectional data from 45 Chinese universities to explore the relationship among perceptions of campus outdoor environments, learning engagement, and college student mental health, and focused on differences among college students in different grades.

**Results:**

Our study revealed the mental health problems of Chinese college students during the COVID-19 pandemic were more severe. The mental health of postgraduates was generally poor, and their risk of depression was higher than that of undergraduates. More importantly, for postgraduates, the direct impact of the perceived campus outdoor environment on their mental health was stronger. For undergraduates, the indirect impact of learning engagement on the effect of the perceived campus outdoor environment on their mental health was stronger.

**Conclusion:**

The results of the study have implications for campus planners, landscape architects, and university planners to pay particular attention to the needs of postgraduates for campus outdoor environments, which is of great significance to improve the overall mental health of students during the COVID-19 pandemic.

## 1. Introduction

There is a growing concern about the mental health of young adults and college students (1–5). Nowadays, college students face academic, interpersonal, financial, and cultural challenges (6). They have relatively high-stress levels and, in turn, are more likely to suffer from mental disorders such as anxiety and depression (5, 7). More and more college students are experiencing depression, anxiety, suicidal thoughts, and other health problems (8–10). The causes of these mental health problems among college students could be attributed to the unique university lifestyles with factors such as academic studies, social stress, financial difficulties, exam anxiety, degree selection, living alone for the first time, and free time management (3, 7, 11, 12). China has 2,852 universities with 37 million students in 2015 (13). Mental health problems caused by various inducements have become a widespread problem among Chinese college students. Significantly during the emergence of COVID-19, the potential for mental health problems among college students has increased due to the wanton spread of the novel coronavirus. (14–18). Adolescents are likely to experience high rates of depression even after the enforced isolation ends. The longer the isolation of students, the greater the risk of health problems (14). Therefore, in the context of the normalization of epidemic prevention and control, more research is needed on the mental health of college students to better understand this problem (19, 20).

With this rising concern of mental health problems, researchers investigate possible solutions from various fields. Among them, the perceived campus environment is one of the important observation factors. The campus environment is similar to the urban environment, which is composed of buildings, open spaces, and walking paths. It is a place for sports, entertainment, learning, and social activities (21). The outdoor environment significantly affects a person’s physical and mental health. The relationship between the natural environment and mental health has been widely theorized and studied by scholars of many subjects (22), such as environmental psychology, geography, urban planning, medicine, and landscape architecture (23). Many studies have shown that green space quality can significantly improve mental health (24) and promote mental restoration among students (25). A more natural outdoor environment and green landscapes can have a positive impact on decreasing stress (26), fostering an overall sense of wellbeing (27), reducing depression (28), and improving cognitive ability and mental health (27, 29–32). The outdoor environment is one of the typical characteristics of the school. Unlike the indoor teaching space, it provides students with open space for physical activities and amusement, socializing with their peers, relishing the beauty of nature, and acquiring outdoor education (33). Although there is no unified definition of the campus outdoor environment, the existing research on campus open space mainly focuses on outdoor green spaces (34) or outdoor venues and facilities that affect sports activities (35). According to its functional nature, campus outdoor activity areas can be mainly divided into two categories: green spaces and outdoor environments for interaction and activity, including sports zones and game equipment, among others (36). On campus, green space is part of the overall student experience (37) and has positive implications for student health (38–40). Studies have also demonstrated that college outdoor green spaces provide an environment for students to release their frustrations, reduce stress levels (41), and improve mental restoration (42), mental and physical health, and quality of life and wellbeing (43, 44). The communication and activity in the outdoor environment of the campus shall engender a salutary effect on the mental health of college students, thus aiding in their recuperation. For instance, open spaces, athletic fields, and exercise amenities proffer college students convivial and reposeful domains for socializing and unwinding (45). The accessibility of green areas in outdoor scenes, the availability of facility structures, the existence and esthetics of sidewalks, and the multiplicity of physical facilities are positively correlated with participation in sports activities (46–48). Those who partake in more esthetically pleasing outdoor activities are more prone to attaining positive psychological dispositions and sound mental wellbeing (49). Sports and other outdoor recreational activities in outdoor communication and activity environments assist students in mitigating academic stress (50). More studies have pointed out that physical activities exposed to the outdoor environment of the campus are more beneficial than indoor physical activities to reduce adverse psychological problems such as tension, confusion, anger, and depression (51, 52). During the COVID-19 pandemic, even if Chinese college students did not have a complete control period, most of their actions were generally limited to campus. Under the long-term closed management, the campus has become the only living place for some college students (53). Finding strategies to strengthen the campus outdoor environment to enhance the quality of life and mental health of college students has essential practical significance. However, most current research on campus environment and mental health in China mainly focuses on walking environments and green spaces (54). Few studies have considered the link between campus outdoor activity environment, campus green spaces, and mental health, especially during the epidemic. There is still some space for research on the perceived campus outdoor environment and the mental health of college students in China (55, 56).

In addition to the subjectively perceived campus environment, learning engagement also plays an essential role in mental health. Learning engagement, a multi-dimensional, multi-level, and evolving concept first proposed by Schaufeli et al. (57) is an important indicator of the quality of students’ learning process (58). It is mainly used to measure the effective time students invest in learning and students’ commitment or dedication to learning activities (59). Some studies have confirmed the relationship between learning engagement and mental health (60–68). The adverse effects of learning engagement and academic performance can in turn affect students’ overall mental health (17, 69). Studies have demonstrated that adults involved in lifelong learning have fewer chronic health problems and are more physically flexible, such as walking more around their city (70). This finding is consistent with the evidence linking lifelong learning participation to better overall life outcomes, especially in physical and mental health (61–74). In addition, some students who have learning problems such as lower learning efficiency, lower learning motivation, and weaker learning persistence are more likely to suffer psychological problems such as depression and anxiety in later life (75, 76).

Research on the correlation between learning engagement and campus environment reveals that for college students, investing time in outdoor settings is an expeditious and efficacious method for enhancing emotional wellbeing (77). Positive emotions can stimulate learning and foster heightened learning engagement (78). Spending more time in green spaces and even watching green landscapes has positive effects on students’ attention and performance (30, 79–81). When students are mentally exhausted, they may become irascible and restless and may have difficulty concentrating or performing simple tasks (22). In the natural environment, students have higher motivation, fun, and participation (82) and can promote the intrinsic motivation of learning engagement and the persistence of learning interest of high school students (83). The social cognitive theory posits that individuals, behaviors, and environments interact and influence each other reciprocally (84). Learning is a social activity. College students spend a significant amount of time in the campus environment on a daily basis. A favorable outdoor campus environment maximizes interpersonal contact and ideational exchange among students, while also augmenting formal indoor learning processes (21). The improvement of communication behavior prompted by the environment and the increase in outdoor activities have enhanced the quality of engagement in learning at school (85). Mental health is complex. The influence of the campus environment on mental health is not direct and is often affected by a variety of factors. For college students, learning engagement is important for students ‘daily life and learning activities. Learning engagement may be a bridge between the campus outdoor environment and mental health. Therefore, learning engagement can be used as a mediator between mental health and the campus outdoor environment. Nevertheless, few studies focus on college students in such studies, and few have explored learning engagement as an intermediary variable. In this context, the complex correlations between perceived campus outdoor environments, learning engagement, and Chinese college students’ mental health have not yet been fully investigated.

Although many previous studies suggest that the campus environment is beneficial to students’ both physical and mental health, the processes and factors behind the correlations between these two factors have not yet been fully investigated. The logical relationship between how the perceived campus outdoor environment affects the mental health of college students is still unclear. There is still a lack of comparative research on the perceived campus outdoor environment, learning engagement, and the mental health of college students. Therefore, it is necessary to incorporate the perceived campus outdoor environment and learning engagement into the mental health impact model of college students for systematic research. Identifying these relationships will not only improve our understanding of the relationship between students’ learning engagement and perceived campus outdoor environment but also enhance our ability to plan and manage the physical environment of Chinese universities, which will improve the overall mental health of Chinese college students.

In addition, it is worth noting that some scholars have paid attention to the heterogeneity of mental health after the academic community generally recognizes the importance of college students’ mental health. However, most studies focus on gender differences (1, 5, 86). There are also some studies on race/ethnicity, LGBTQ+ identity, college generation identity, social economy (87), parents with higher education (88, 89), STEM major, sector, and GPA (self-report, 4.0 points), which confirmed that these variables may be associated with mental health (87, 90–95). Although some scholars believe that over time, the rate of depression in college students has increased (96, 97), there are relatively few studies on the differences in the mental health of Chinese college students of different grades. Therefore, it is necessary to consider the differences in the mental health of students of different grades.

Thus, our study focuses on the problem of the perceived campus outdoor environment and mental health of Chinese college students and analyzes the differences among college students of different grades. We aim to better understand the relationship between the perceived campus outdoor environment, learning engagement, and the mental health of college students. In this way, we can put forward targeted suggestions for the improvement of the mental health of students of different grades. Our research mainly raises the following questions:

1. Are there grade differences among the mental health, perceived campus outdoor environment, and learning engagement of Chinese university students?
2. Whether perceived campus outdoor environment and learning engagement are associated with self-efficacy?
3. Are there grade differences in the relationship between perceived campus outdoor environment and learning engagement with the mental health of college students?

## 2. Materials and methods

### 2.1. Study population

The survey was conducted from 1 October 2021 to 30 January 2022. All investigations were carried out during the morning on weekdays. Our horizontal section data were collected using a questionnaire of 1,261 students from 45 universities in China. In order to enhance the representativeness of the sample, the selection of the sample was based on a sampling principle that prioritized diversity in geographical location, university classification, and region, among other factors. All the participants completed an online survey and answered specific related questions, including the evaluation of their mental health, campus outdoor environmental perceptions, and learning engagement. The questionnaire star survey platform^1^ was our electronic questionnaire release. Prior to the formal distribution of the questionnaire, we conducted a pilot study with 10 students and engaged in in-depth discussions regarding the rationality of the questions in the questionnaire. Based on their feedback, we made appropriate revisions to the questionnaire. To ensure the validity of the data, we set up strict requirements for the validity of the questionnaire and removed the invalid questionnaires that were randomly filled out and maliciously received rewards to obtain a truly effective questionnaire. The specific measures include: we have multiple restrictions and multiple screening in terms of questionnaire access, question setting, real-name lottery, repeated IP screening, and setting the shortest answer time, and the deans, teachers, and other leaders of each school and college assist the questionnaire. The survey objects were undergraduate, postgraduate, and doctoral students. The survey covered 45 universities, 20 provinces, and 30 cities in China. Under the effective organization of university leaders, we obtained a total of 1,236 valid samples, with an effective rate of 98% (see Table 1 for the sample statistics).

**Table 1:** The sample demographics.

Demographics	*N*	%
Grade freshman	440	35.6
Sophomore	213	17.2
Junior	206	16.7
Senior	201	16.3
Master	158	12.7
Doctor	18	1.4
Gender		
Male	553	45.16
Female	683	54.84
Subject		
Liberal arts	313	6.42
Sciences	923	8.19
Monthly living expenses		
Less than 1,000 yuan	118	9.5
1,000–2,000	762	61.7
2,000–3,000	270	21.8
3,000–5,000	60	4.9
5,000–8,000	14	1.1
More than 8,000	12	0.9
Self-assessment of health		
Very bad	23	1.9
Bad	82	6.6
General	530	42.9
Better	442	35.8
Very good	159	12.9
Father’s level of education		
Elementary school and below	124	10
Junior high school	387	31.3
High school or technical secondary school or technical school	326	26.4
Junior university	169	13.7
Undergraduate	197	15.9
Master’s degree or above	33	2.7
Mother’s level of education		
Elementary school and below	221	17.9
Junior high school	377	30.5
High school or technical secondary school or technical school	308	24.9
Junior university	161	13
Undergraduate	142	11.5
Master’s degree or above	27	2.2

### 2.2. Measurement

#### 2.2.1. Dependent variable: Mental health of college students

Mental health issues have received widespread attention from many scholars since the novel coronavirus pandemic (98). According to previous studies, depression assessment has become the most important indicator of mental health. According to the World Health Organisation (WHO), depression is defined as a common mental illness that usually leads to low mood, loss of interest, feelings of inferiority, lack of energy, and inattention (99). The impact of the novel coronavirus pandemic on college students’ mental health is particularly prominent and severe in terms of depression (100). Therefore, in the emotional factors affecting the mental health of college students, this study mainly focuses on depression. The depression we studied was assessed by the WHO-5 Happiness Index (WHO-5), a tool used to assess participants’ mental health benefits, which is highly effective and reliable in screening for depression (101–105). The Happiness Index Scale includes five positive emotional items: (1) feeling happy and comfortable, (2) feeling calm and relaxed, (3) feeling energized, (4) waking up feeling awake and well-rested, and (5) everyday life is full of exciting things. Participants were asked about the frequency of these positive emotions in the recent 2 weeks. A score ranged from 6 to 0. Less than 13 points indicate depression. The higher score indicates better mental health.

#### 2.2.2. Independent variable: Perceived campus outdoor environment

The campus outdoor environment in our study mainly referred to students’ subjective perceptions of the campus environment. Although there are differences in the specific indicators of the perception of the outdoor environment of the campus, the standard formulas of accessibility, convenience, esthetics, safety, satisfaction, and comfort are applicable to outdoor space (106–108). Our research combined existing findings and divided the campus outdoor environments into two sections consisting of a total of nine issues. Section one (campus green spaces) contained four questions, including greening comfort, reasonable layout, beautiful scenery, and plant diversity. In the second section (campus outdoor activity environment), we referenced the perceptual environmental scale developed by Mujahid et al. (109). This section contained five questions, including sufficient outdoor spaces, reasonable layout, complete facilities, reasonable lighting design, and physical exercise places and equipment. In each item, the response ranged from 1 to 5 (1 = completely disagree, 2 = disagree, 3 = neutral, 4 = agree, 5 = completely agree), and the higher score indicated the higher the respondents’ recognition of all aspects of the campus outdoor environment.

#### 2.2.3. Intermediary variables: Learning engagement

Our research referred to the schoolwork engagement scale (110, 111), and used four items to measure learning engagement, including “I feel energized when I study,” “I am enthusiastic about my studies,” Time flies when I’m studying,” and “I feel happy when I devote myself to study..” All the items were rated on a 5-point scale (1 = never, 2 = rare, 3 = sometimes, 4 = often, 5 = always). The higher scores suggest a higher level of learning engagement.

#### 2.2.4. Control variables

In the conceptual model of this article, gender, parental education level, students’ education level, subject, and monthly expenditure were included as control variables. Education levels were assigned as follows: 1: freshman; 2: sophomore; 3: junior; 4: senior; 5: Master1; 6: Master2; 7: Master3; 8: 1st Year PhD student; 9: 2nd Year PhD student; and 10: 3rd Year PhD student and above. The item of parental education level was scored on a scale of 1 to 7 (1: elementary school and below, 2: junior high school, 3: senior high school, technical secondary school, and technical school, 4: junior college, 5: bachelor, 6: master, and 7: doctor). The subjects are as follows: 1. Humanities and 2. Science. The item of monthly expenditure was scored on a scale of 1 to 6 (1: less than 1,000 yuan, 2: 1,000–2,000 yuan, 3: 2,000–3,000 yuan, 4: 3,000–5,000 yuan, 5: 5,000–8,000 yuan, and 6: more than 8,000 yuan).

#### 2.2.5. Statistical analysis

This study discussed the relationship between the perceived campus outdoor environment, learning engagement, and college students’ mental health. We validated the multi-factor analysis of all measurement models in the conceptual model. The result showed that all the measurement models’ compositional reliability was greater than 0.6; the average variance extraction was greater than 0.5; the factor load of the observed variables was greater than 0.6; the reliability coefficient was greater than 0.36 (112), and all the measurement models had good reliability and validity. Relevant studies have shown that the X2/degrees of freedom ratio is partially affected by the sample size (113). As a large sample size model with a sample size greater than 750, the x2/df in our study can be slightly relaxed. Our x2/df is 3.545, below the recommended level of 5 (114). Other fit quality indexes (GFI > 0.9, AGFI>0.9, RMR > 0.9, NFI > 0.9, CFI > 0.90, TLI > 0.90, RMSER<0.08) achieved the criteria, which showed that the model was fit (Table 2).

**Table 2:** The fitness fitting index of the full sample model.

	*X*^2^/df	GFI	AGFI	CFI	RMR	NFI	TLI	RMSEA
The full sample model	3.545	0.948	0.932	0.978	0.043	0.969	0.973	0.045
Ideal standard	<5	>0.9	>0.9	>0.9	<0.05	>0.9	>0.9	<0.08

After dividing all observed variables in our study into high and low groups based on the 27th and 73rd percentiles and conducting t-tests (115), we found significant differences between the high and low groups. Therefore, all variables exhibit good discriminant validity. Given that the sample size for our study is 1,236 (>1,000) and the sample mean is approximately normally distributed, we utilized the asymptotically distribution-free (ADF) statistical method. Consequently, the sample data in our study is appropriate for SEM analysis.

Multiple-factor confirmatory analysis was conducted on the measurement model of the conceptual model. The composite reliabilities of the five measurement models, namely campus outdoor environment, campus green space, campus outdoor activity environment, learning engagement, and mental health, were 0.971, 0.951, 0.941, 0.927, and 0.948, respectively, all exceeding the standard of 0.7. The average variance extracted (AVE) for each measurement model were 0.792, 0.828, 0.763, 0.761, and 0.784, all exceeding the standard of 0.5 (116). The standardized factor loading and SMC of the observed variables were all greater than or close to the standards of 0.6 and 0.36 (Table 3). All measurement models exhibited good reliability and validity and were suitable for structural equation modeling analysis.

**Table 3:** Variable validity and reliability test.

Measurement model (CFA)	Observed variable	Model parameter estimates				Convergent validity			
		Non-standardized factor loadings	Standard error (S.E.)	C.R. *t*-value	*P*	The standardized factor loadings	SMC	C.R.	AVE
Campus outdoor environment	Campus outdoor activity environment	1.000				0.943	0.889	0.971	0.792
	Campus green spaces	0.858	0.046	18.761	***	0.822	0.676		
Campus green spaces	GS1	1.003	0.022	45.137	***	0.905	0.819	0.951	0.828
	GS2	1.025	0.021	48.448	***	0.939	0.882		
	GS3	1.027	0.019	53.172	***	0.927	0.859		
	GS4	1.000				0.867	0.752		
Campus outdoor activity environments	AE1	1.058	0.027	38.897	***	0.884	0.781	0.941	0.763
	AE2	1.058	0.026	40.784	***	0.908	0.824		
	AE3	1.107	0.026	41.952	***	0.922	0.850		
	AE4	1.026	0.029	34.940	***	0.823	0.677		
	AE5	1.000				0.827	0.684		
Learning engagement	LE1	1.050	0.025	42.288	***	0.925	0.856	0.927	0.761
	LE2	1.079	0.026	41.652	***	0.916	0.839		
	LE3	0.943	0.028	34.199	***	0.810	0.656		
	LE4	1.000				0.831	0.691		
Mental health	MH1	1.000				0.893	0.797	0.948	0.784
	MH2	1.070	0.018	60.207	***	0.904	0.817		
	MH3	1.093	0.019	58.183	***	0.936	0.876		
	MH4	1.058	0.031	34.462	***	0.863	0.745		
	MH5	1.106	0.030	37.162	***	0.909	0.826		

***represents significant at the 1% level.

## 3. Results

### 3.1. Descriptive statistics

The depression rates among different grades are shown in Table 4. It shows that most Chinese college students’ mental health is general during the COVID-19 pandemic. In general, 27.3% of the respondents suffer from depression. The incidence of depression in freshmen is 20.0%, in sophomores to seniors is 30.8%, and in postgraduates is 33.0%. Obviously, the mental health of freshmen is the best and that of postgraduates is the worst. By and large, the higher the grade, the worse the mental health and the higher the incidence of depression among college students.

**Table 4:** The comparison of depression prevalence among different groups.

Different groups of college students		Depression percentage %
Grade	Freshmen	20.0
	Sophomore to senior students	30.8
	Postgraduate	33.0

The descriptive statistics of variables are shown in Table 5. The overall evaluation of college students in campus outdoor environments is relatively good. The mean values of students’ satisfaction with campus green spaces are higher than 3.7, and the mean values of satisfaction with campus outdoor activity environment are higher than 3.4. Meanwhile, the mean values of students’ satisfaction with outdoor activity environments are lower than that of campus green spaces.

**Table 5:** Variable descriptive statistics.

	Observed Variables		All		Grades (mean)		
			Mean	S.D.	Freshmen	Sophomore to senior students	Postgraduate
The mental health of college students	MH1		3.44	1.26	3.71	3.30	3.27
	MH2		3.28	1.33	3.60	3.12	3.07
	MH3		3.29	1.32	3.60	3.13	3.08
	MH4		3.13	1.38	3.41	2.98	2.98
	MH5		3.24	1.37	3.54	3.06	3.10
Campus environment	Campus outdoor activity environment	AE1	3.53	1.02	3.34	3.52	3.66
		AE2	3.51	1.00	3.29	3.48	3.69
		AE3	3.48	1.02	3.19	3.48	3.65
		AE4	3.45	1.07	3.21	3.42	3.67
		AE5	3.56	1.03	3.34	3.53	3.75
	Campus green spaces	GS1	3.83	0.93	3.74	3.77	3.99
		GS2	3.76	0.91	3.61	3.72	3.94
		GS3	3.81	0.93	3.64	3.77	3.98
		GS4	3.75	0.97	3.59	3.71	3.93
Learning engagement	LE1		3.63	0.82	3.25	3.57	3.99
	LE2		3.63	0.85	3.25	3.54	4.01
	LE3		3.79	0.84	3.48	3.72	4.11
	LE4		3.75	0.87	3.50	3.65	4.10
Control variable	Father education level		3.03	1.34	2.88	2.98	3.24
	Mother education level		2.77	1.34	2.61	2.70	3.01
	Cost		2.30	0.86	2.28	2.25	2.41
	Education		2.71	1.80	1.00	2.98	6.03
	Gender		1.55	0.50	1.39	1.64	1.64
	Subject		1.75	0.43	1.91	1.63	1.76

The higher the grade, the better the learning engagement of students. In the control variables, the average education level for college students is sophomore, the father’s education level is above senior high school, the mother’s education level is above junior high school, and the monthly expenditure of college students is 1,000–2000 yuan or more.

### 3.2. Analysis based on the models of full sample

To verify if there is a mediating effect of learning engagement on the impact of campus outdoor environment and college students’ mental health, this study employs three methods, the Bias-Corrected Confidence Interval (CI) method using Bootstrap, the Percentile CI method, and the *Z*-test method. The use of all three methods ensures the rigor of the study (117). In the path of the influence of campus outdoor environment on mental health, under the 95% confidence interval, the lower-to-upper values of the total effect and indirect effect of the two test methods do not include 0, and the *Z*-value is greater than 1.96, indicating that the mediating effect exists, that is, learning engagement has a mediating effect in the path of campus outdoor environment affecting mental health. The lower to upper values in the direct effect does not include 0, and the Z-value is greater than 1.96, indicating that the intermediary is partial. The campus outdoor environment positively affects the mental health of college students through the partial intermediary of learning engagement. The statistical details of the intermediary effect test are shown in Table 6.

**Table 6:** The mediating effect test of learning engagement in the influence path of campus outdoor environment on college students’ mental health.

Variables	Point estimates	Product of coefficients		Bootstrap			
				Bias-corrected 95% CI		Percentile 95% CI	
		SE	*Z*	Lower	Upper	Lower	Upper
Campus outdoor environment–Mental health	**Total effect**						
	0.603	0.051	11.823	0.518	0.686	0.518	0.686
	**Direct effect**						
	0.282	0.052	5.423	0.199	0.371	0.197	0.370
	**Indirect effect**						
	0.321	0.039	8.231	0.261	0.391	0.260	0.390

**Figure 1:**
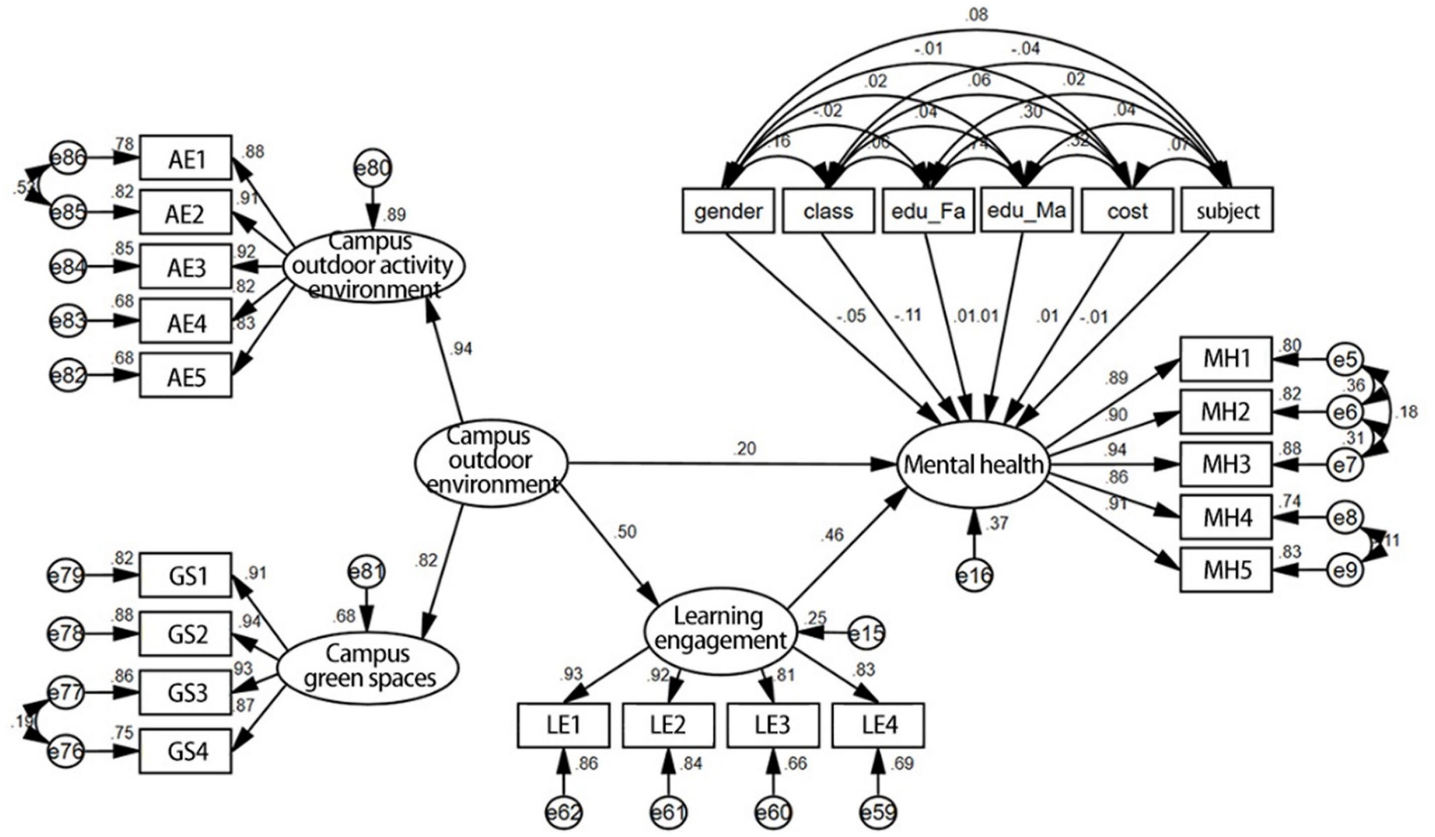
Standardized path diagram for the whole sample model.

Model-fitting results for the entire sample are shown in Table 7 and Figure 1. After controlling for education, parental education level, monthly expenditure, and gender, the total effect value for perceived campus outdoor environment and learning engagement on the mental health of college students are 0.43 and 0.46 in sequence. The effect value for the perceived campus outdoor environment on the learning engagement of college students is 0.50. The direct and indirect values for the perceived campus outdoor environment on the mental health of college students are 0.20 and 0.23 in sequence. College students’ mental health is impacted in direct and indirect ways by the perceived campus outdoor environment, indicating that there may be intermediary variables on this path. The intermediary effect value of learning engagement is 0.23. It shows that the positive impact of the perceived campus outdoor environment on college students’ health is related to learning engagement.

**Table 7:** The standardized effect value of the full sample model.

Independent variable	Intermediate variable	Dependent variable		
	Learning engagement	Mental health		
		Total effect	Direct effect	Indirect effect
Campus outdoor environment	0.50***	0.43***	0.20***	0.23***
Learning engagement	–	0.46***	0.46***	–

***means significant at the 1% confidence level.

### 3.3. Comparison of model differences among different income groups

**Figure 2:**
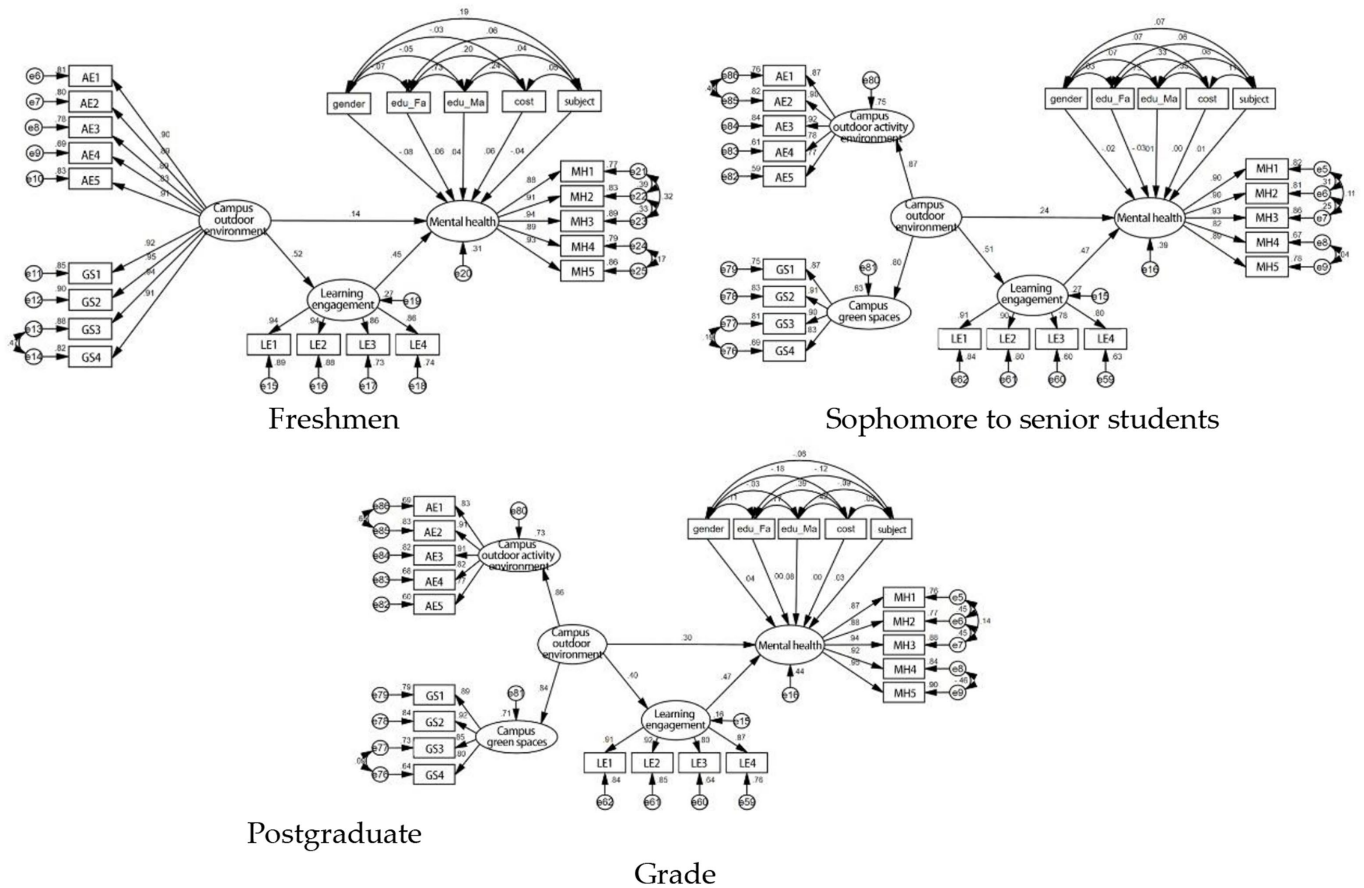
Comparison of standardized road maps of different university student population models.

Table 8 and Figure 2 compared the model fitting results based on samples of different grades of college students. The data of freshmen, sophomore to senior students, and postgraduate groups were substituted into the model for group comparison. The fitting result of the freshmen group model showed that the fitting result of the second-order measurement model of the campus outdoor environment was faulty, and the factor loading of the campus outdoor activity environment exceeded one. Therefore, the freshmen group model was modified to integrate the measurement model of the campus green spaces and outdoor activity environments into one variable, and thereafter, the model was re-fitted. This resulted in an adequately fitting result, as presented in Figure 2.

**Table 8:** Comparison of path coefficients for different university student groups model.

	Argument		Intermediate variable	Dependent variable		
			Learning engagement	Mental health		
				Total effect	Direct effects	Indirect effects
Grade	Freshmen	Campus outdoor environment	0.52***	0.37***	0.14***	0.23***
		Learning engagement	–	0.45***	0.45***	–
	Sophomore to senior students	Campus outdoor environment	0.51***	0.48***	0.24***	0.24***
		Learning engagement	–	0.47***	0.47***	–
	Postgraduates	Campus outdoor environment	0.40***	0.49***	0.30***	0.19***
		Learning engagement	–	0.47***	0.47***	–

***represents significance at the 1% level.

The comparison of the fitting results of different grades shows that the mental health of college students in different grades is significantly positively affected by the perceived campus outdoor environment and learning engagement. The direct and indirect effect values of perceived campus environment on the mental health of freshmen are 0.14 and 0.23, respectively, and the total effect was 0.37. The effect value of learning engagement on the mental health of freshmen is 0.45. The effect value of the perceived campus outdoor environment on freshmen’s learning engagement is 0.52. The direct and indirect effect values of the perceived campus outdoor environment on the mental health of sophomore to senior students are both 0.24, and the total effect is 0.48. The effect value of learning engagement on the mental health of sophomore to senior students is 0.47. The effect value of the perceived campus outdoor environment on sophomore to senior students’ learning engagement is 0.51. The direct and indirect effect values of the perceived campus environment on the mental health of postgraduates are 0.30 and 0.19, respectively, and the total effect is 0.49. The effect value of learning engagement on the mental health of postgraduates is 0.47. The effect value of the perceived campus outdoor environment on postgraduates’ learning engagement is 0.40. This result indicated that the positive effect of the perceived campus outdoor environment on the mental health of students of different grades needs to be realized by improving their learning engagement.

## 4. Discussion

Our study explored the mechanism of the effects of perceived campus outdoor environment and learning engagement on the mental health of college students during the COVID-19 pandemic. Furthermore, we compared the differences in the mechanism among college students of different grades. Our aim was to propose suggestions and feasible measures from the perspective of the outdoor environment and college management to improve the mental health of college students of different grades.

Our study found that the mental health problems of Chinese college students during the COVID-19 pandemic were more severe, and 27.3% of college students suffered from depression. In consequence, finding effective ways to intervene and improve the mental health issues of this special group was worthy of attention. More importantly, there were significant differences in mental health among different groups of college students. The mental health of college students deteriorates with the increase in grades. The mental health of postgraduates was generally poor, and the risk of depression was higher than that of undergraduates.

We confirmed that the outdoor environment on campus is positively correlated with the mental health of college students, which is consistent with the study before the novel coronavirus epidemic (40, 54, 118–121). Especially during the COVID-19 epidemic, when colleges generally implemented closed management, college students stay longer on campus. At this time, the campus outdoor environment may play a more important role in the mental health of college students. From the existing literature, more scholars have focused on the impact of the epidemic on students ‘mental health (122), but there is a lack of attention to the influence of the perceived campus outdoor environment on college students’ mental health. Finding factors that can improve the mental health of college students to intervene may be more beneficial to solve this problem. Therefore, our research conclusion is a supplement to the current literature and a useful reference for how to improve the mental health of college students.

Although our research was aimed at the specific place of the campus environment, many scholars have studied other environments from a broader perspective. Their findings also confirmed the impact of human settlements on mental health (123). Some scholars also pointed out that special attention should be paid to vulnerable groups such as the older adults people and the poor (124). More relevant studies have studied the heat-related impacts on daily functions (125) and incorporated future climatic uncertainties into the consideration of the built environment (126). Some studies compared how outdoor design features are used by students with how these features are reported as being used (127). Planners should put forward targeted suggestions for environmental transformation and construction. Our study confirmed the correlation between campus outdoor environments and mental health, especially among postgraduates in China. Therefore, in the subsequent campus design and planning, special attention should be paid to the construction of the campus greening environment, enriching the content of outdoor activities and making a more reasonable layout design for the greening landscape and activity places of outdoor space.

Furthermore, we found that the perceived campus environment affects college students’ mental health through the mediating effect of learning engagement. Some studies have confirmed the link between campus environment and learning engagement (82). Our research further confirmed that the increase in the natural environment on university campuses and the improvement of outdoor activity environment and infrastructure will have a positive impact on students’ learning engagement during the COVID-19 pandemic. Existing research has confirmed that learning engagement is closely related to college students’ mental health problems (60), and our research also proved this. Therefore, to improve the mental health of college students, it is necessary to focus on various aspects, including the quality of the campus outdoor environment and students’ engagement in learning.

On the whole, learning engagement played an important mediating role in the path of campus outdoor environment affecting the mental health of college students, and its role was even stronger than the direct impact of campus outdoor environment on mental health. This showed that the impact of the campus environment on college students’ mental health was mostly achieved by improving learning engagement. Learning engagement was an important predictor of the mental health of college students, not only because it had a high impact on mental health but also because it was also an important intermediary variable for external support factors such as campus outdoor environment.

More importantly, we found that the influence of campus outdoor environment and learning engagement on the mental health of different groups of college students was different. For postgraduates, the direct impact of the campus outdoor environment on mental health was greater than that of undergraduates, while the intermediary impact of learning engagement on mental health was slightly lower than that of undergraduates. In addition, some perceived campus outdoor environment measurements of higher-grade students are higher than lower-grade students. This difference may be related to the different times students enter school. Compared with the lower-grade group, the higher-grade group tends to have a longer campus life, higher recognition, and emotional dependence on the campus environment, so their evaluation of the campus outdoor environment is relatively high. It also helps us to deeply understand and explain our research conclusion, that is, the mental health of higher-grade students who live longer in school is more affected by the campus outdoor environment.

Consequently, to improve the mental health of college students, it is particularly necessary to attach special attention to the needs of groups with relatively poor mental health and put forward specific opinions and strategies. First, the improvement of the campus outdoor environment is extremely important for students of different grades. Campus outdoor environment plays a positive role in promoting the mental health of college students. Creating a campus outdoor environment that promotes positive emotions can effectively alleviate stress among college students, thereby improving their mental health. The improvement of the campus environment needs to start from various aspects. Postgraduates’ mental health is lower than undergraduates, and their depression is relatively higher than undergraduates. Therefore, postgraduates need special attention in campus groups. Their mental health is more directly affected by the campus outdoor environment. Improving the campus outdoor environment can greatly improve the mental health of this group. As a consequence of that, schools should pay special attention to the special requirement of postgraduates for campus outdoor environment, and improve the recognition of campus green spaces and outdoor activity environment. Moreover, actively creating outdoor facilities and activity spaces that facilitate communication and interaction, rather than solely considering their availability, can effectively enhance the mental health of graduate students from multiple perspectives.

Previous research on the relationship between campus outdoor environments and learning engagement remains inadequate. The underlying processes and factors linking campus outdoor environments, learning engagement, and the mental health of college students have yet to receive sufficient study. Our research incorporates the perception of the campus outdoor environments and learning engagement into a model assessing the impact on the mental health of college students. This supplements existing research in this area and provides a new avenue for improving the mental health of college students. Furthermore, we addressed the issue of heterogeneity in mental health. We found that the influence of the perceived campus environment and learning engagement on the mental health of college students differs across different grades. This facilitates targeted campus design interventions for students of varying grades to improve their mental health. Nevertheless, this research has a few shortcomings. First, our research is based on cross-sectional data, which is difficult to accurately clarify the causal relationship among perceived campus outdoor environment, learning engagement, and college students’ mental health, and requires follow-up longitudinal studies. Second, we have not achieved complete random sampling, thus, there will be a certain deviation in the representativeness of the samples. Third, the survey scope and the number of the sample are limited. This research could not represent all campus outdoor environments in China because only a few colleges are selected for an in-depth survey. In future, more empirical studies need to be conducted. In addition, the evaluation of the campus outdoor environment is subjective, and cannot represent the objective campus environment in China. To better evaluate the campus environment in China, future investigations on this subject may consider objective measurements. In addition, our research was conducted during a period of normalization of the epidemic in China. This period was relatively stable and there were no point or concentrated outbreaks. Basically, there were no special circumstances such as being locked in dormitories among our interviewees. But despite this, there are still differences between different specific situations. Finally, more nuanced research is needed to explore whether the campus outdoor environment influences learning engagement through its impact on student communication and teacher–student interactions. In addition, further research is required to investigate whether campus outdoor activity environments can promote physical activity among students, leading to increased learning engagement and ultimately, improved mental health.

## 5. Conclusion

The results of this study reported that the mental health problems of Chinese college students during the COVID-19 pandemic were more severe. The mental health of college students deteriorates with the increase in grades. The mental health of postgraduates was generally poor, and the risk of depression was higher than that of undergraduates.

This study found that the campus outdoor environment affects the mental health of college students through the mediating role of learning engagement. More importantly, we found that for postgraduates, the direct influence of the campus outdoor environment on their mental health was stronger. For undergraduates, the indirect impact of learning engagement in the effect of campus outdoor environment on their mental health was stronger.

Therefore, in future construction and transformation of campuses, it is necessary to put forward different environmental transformation strategies according to the different grades of college students. We should especially pay attention to the needs of postgraduates with poor mental health for the campus outdoor environment, which is of great significance to improving the overall mental health and the quality of life of students during the COVID-19 pandemic.

## Data availability statement

The original contributions presented in the study are included in the article/supplementary material, further inquiries can be directed to the corresponding author.

## Author contributions

NS and ZZ: conceptualization, resources, and writing—review and editing. NS: writing—original draft preparation, software, funding acquisition, and supervision. NS, ZZ, and WL: methodology, validation, data curation, visualization, and project administration. All authors have read and approved the final manuscript and contributed to the article and approved the submitted version.

## Funding

This research was funded by the National Social Science Found of China, grant number 21BRK020.

## Conflict of interest

The authors declare that the research was conducted in the absence of any commercial or financial relationships that could be construed as a potential conflict of interest.

## Publisher’s note

All claims expressed in this article are solely those of the authors and do not necessarily represent those of their affiliated organizations, or those of the publisher, the editors and the reviewers. Any product that may be evaluated in this article, or claim that may be made by its manufacturer, is not guaranteed or endorsed by the publisher.

## Footnotes

^1^ https://www.wjx.cn/app/survey.aspx
